# Forcing Convection to Aggregate Using Diabatic Heating Perturbations

**DOI:** 10.1029/2021MS002579

**Published:** 2021-10-09

**Authors:** Beth Dingley, Guy Dagan, Philip Stier

**Affiliations:** ^1^ Atmospheric, Oceanic and Planetary Physics University of Oxford Oxford UK; ^2^ Now at The Hebrew University of Jerusalem Jerusalem Israel

**Keywords:** self‐aggregation, convective organization, tropical convection, aerosol‐cloud interactions

## Abstract

Tropical deep convection can aggregate into large clusters, which can have impacts on the local humidity and precipitation. Sea surface temperature (SST) gradients have been shown to organize convection, yet there has been little work done to investigate the impact of diabatic heating perturbations in the atmosphere on the aggregation of convection. Here we investigate how anomalous diabatic heating of the atmospheric column, through an idealized aerosol plume, affects the existence and mechanisms of convective aggregation in non‐rotating, global radiative‐convective equilibrium simulations. We show that the aerosol forcing has the ability to increase the degree of aggregation, especially at lower SSTs. Detailed investigation shows that the diabatic heating source incites a thermally driven circulation, forced by the shortwave perturbation. The increase in aggregation is caused in part by this circulation, and in part by the longwave heating anomalies occurring due to the surface convergence of moisture and convection. At higher SSTs, longwave feedbacks are crucial for the aggregation of convection, even with the shortwave heating perturbation. At lower SSTs, convection is able to aggregate with the shortwave perturbation in the absence of longwave feedbacks. These perturbations provide a link to studying the effects of absorbing aerosol plumes on convection, for example during the Indian monsoon season. We argue that, as there is aggregation for plumes with realistic aerosol absorption optical depths, this could be an analogue for real‐world organization in regions with high pollution.

## Key Points

- Can increase the degree of aggregation by using a shortwave radiative heating perturbation
- Radiative heating perturbation incites a global thermally driven circulation
- Shortwave radiative heating perturbation can aggregate convection at lower sea surface temperatures, even without typically crucial longwave feedbacks

## 1 Introduction

Moist deep convection is important for distributing heat, moisture, and momentum globally, however, its intensity and evolution depend on its environment. Convection can be defined as organized when multiple convective cores group together to form large clusters. Convective organization can take many forms, such as squall lines, mesoscale convective systems or supercell clusters (Tobin et al., 2012). Organized convection contributes a significant amount of the total tropical precipitation and cloudiness. Whilst the physical processes underlying the organization of convection have been studied a great deal, there are still many gaps in our understanding of this phenomenon, including the mechanisms driving organization (Tobin et al., 2013).

A common method for studying tropical deep convection is modeling in a radiative‐convective equilibrium (RCE) setup. RCE is the equilibrium state the atmosphere would reach in the absence of lateral energy transport. In this state, the equilibrium is reached by a balance between the convective and radiative processes (Held et al., 1993; Manabe & Wetherald, 1967). Whilst this is an idealized setup, it provides an acceptable simplification of the tropical atmosphere on large scales (Jakob et al., 2019) and has been used to study convection for many years (e.g., Held et al., 1993; Manabe & Wetherald, 1967; Tompkins & Craig, 1998, and others). Models running in the RCE framework generally remove all rotation and make both the solar insolation and sea surface temperature (SST) constant. Whilst these are simplifications of the Earth's atmosphere, they work as a reasonable representation of the tropics, due to the small tropical Coriolis forces and large ocean heat capacity. This setup is useful for investigating some of the key physical drivers of convection such as humidity, radiative and surface fluxes, and convectively driven circulations (e.g., Becker et al., 2017; Popke et al., 2013).

A specific convective organization phenomenon that can occur when running a model in RCE is convective self‐aggregation. This is the transition from scattered convection to one or several organized convective clusters in the absence of external forcing (Bretherton et al., 2005). Although this was originally found to occur in 2D cloud‐resolving models (CRMs) (Held et al., 1993), it has since been observed in many different model configurations in an RCE setup, such as 3D CRMs, global and regional models with parameterized convection and global models with super‐parameterized convection (Wing, 2019). Aside from being a reorganization of the convection in a model, convective self‐aggregation also impacts the horizontal domain‐mean state of a simulation. Whilst some of the effects of aggregation are still disputed, it is generally agreed that, once convection aggregates, there is domain‐mean drying of the relative humidity profile, free‐tropospheric warming, and more radiation is emitted to space (e.g., Bretherton et al., 2005; Muller & Held, 2012). Self‐aggregation also often leads to a decrease in the high‐cloud fraction and the mid‐level cloudiness (Cronin & Wing, 2017). The response from low clouds is less clear ‐ some studies show an increase in the low cloud fraction (Stein et al., 2017; Tobin et al., 2013), while others show a decrease (Tobin et al., 2012). Precipitation is also often seen to increase when the convection has aggregated (Cronin & Wing, 2017). More locally, self‐aggregation causes an increase in intense precipitation in the clusters, with increased drying outside of the clusters. This leads to a broadening of the moisture distribution. The combination of these effects implies that self‐aggregation has the potential to change the climate, thus emphasizing the need for a better understanding (Wing, 2019).

It has been robustly shown that interactions between longwave radiative feedbacks, moisture, and convection are essential for triggering and maintaining aggregation (e.g., Coppin & Bony, 2015; Muller & Held, 2012; Yang, 2018a). This physical feedback relies on strong clear‐sky longwave radiative cooling away from deep convective areas, which helps to develop a circulation which advects moist‐static energy up‐gradient to the moist convecting region (Muller & Held, 2012). There is an increasing body of work suggesting the boundary layer is especially important for feedbacks involved in convective aggregation (e.g., Muller & Bony, 2015; Naumann et al., 2017; Yang, 2018a, 2018b). Yang (2018a) showed through mechanism denial experiments, that removing longwave feedbacks in the boundary layer is enough to suppress aggregation, whilst removing the free‐tropospheric feedbacks alone still enabled the convection to aggregate, verifying that the boundary layer is important for convective self‐aggregation. There has been some dispute over the remaining physical drivers of aggregation, with different studies giving opposing results. Some proposed positive feedbacks include the virtual effects of water vapor (Yang, 2018a), and the wind‐induced surface heat exchange (WISHE) feedback (Coppin & Bony, 2015). The shortwave radiative feedbacks have been found to have differing signs. Muller and Held (2012) found that interactive shortwave fluxes were not essential in triggering aggregation and that shortwave fluxes opposed the formation of aggregation. They hypothesize that this is due to increased shortwave cooling in the cloudy regions. Other studies, such as Wing and Emanuel (2014) and Holloway and Woolnough (2016) have contradicted this result, finding that there is reduced shortwave absorption in the dry regions, due to the absorption of water vapor. It has also been shown that the dominant mechanisms can depend on the model parameters chosen, such as SST, horizontal grid resolution, or the representation of convection (Coppin & Bony, 2015; Muller & Held, 2012; Wing, 2019).

Shamekh et al. (2020) investigated the concept of forced convective aggregation. They used a CRM with fixed SSTs, and with SST ’hot‐spots’ of between 3 and 5 K and looked at how this forced the aggregation and whether it differed from self‐aggregation. They noted an acceleration in the formation of aggregation, especially for warmer/larger hot‐spots. They also saw that the inclusion of a hot‐spot allowed simulations to aggregate at temperatures that they previously would not. Convective instability over the hot‐spot forces a large‐scale circulation and this increases the drying from subsidence outside of the anomaly.

Khairoutdinov and Emanuel (2010) found that, in their simulations, convective aggregation exhibits hysteresis: once convection has aggregated, it remains so even if the SST is subsequently dropped below their critical threshold temperature. Muller and Held (2012) also found hysteresis in their simulations, including when key mechanisms required to form aggregation are removed after the simulation has aggregated. In most convective self‐aggregation studies this hysteresis feature is seen, however, Shamekh et al. (2020) found that the aggregation that exists due to the SST hot‐spots does not exhibit hysteresis, with convection disaggregating again once the hot‐spot is removed.

Convective self‐aggregation is formed by similar mechanisms to observed organized convection (such as mesoscale convective systems) however the link between the simulated and observed aggregation remains unclear and is one of the key questions in the study of convective self‐aggregation. Many of the processes organizing the convection in these idealized studies are exhibited on a much larger scale in the formation of the Madden‐Julien Oscillation (MJO) (Arnold & Randall, 2015), hence this is a possible real‐world analogue of self‐aggregation. Another avenue that has not yet been explored is the ability of strong pollution plumes to influence the organization of convection. Roeckner et al. (2006) have shown that plumes of absorbing aerosols in the tropics can heat the atmosphere, induce thermally direct circulations and locally increase moisture convergence and precipitation. Further, in Dagan et al. (2019) an absorbing aerosol plume was introduced into the tropics of an aquaplanet model; the plume incited a large‐scale thermally driven circulation and the authors conclude that this could promote convective aggregation in the tropics.

In this study, we aim to utilize the results in Dagan et al. (2019) to investigate the impact of external diabatic heating on convective organization. We introduce diabatic heating based on an idealized plume model, which is representative of aerosol radiative perturbations. The plume used (see details in Section 2.3) creates a strong local shortwave radiative heating perturbation. This creates a large‐scale thermally driven circulation, which encourages moisture convergence near the surface and divergence in the upper atmosphere (Dagan et al., 2019). We hypothesize that this, in turn, will encourage convection to form and hence convective aggregation will form around the plume forcing. Henceforth, when this process is discussed, it is referred to as ‘forced aggregation’ and it should be noted that this differs from spontaneously occurring convective self‐aggregation. In particular, we aim to answer the following questions:

1. Can the inclusion of a diabatic heating perturbation increase the degree of aggregation in GCM RCE simulations?
2. How do the mechanisms driving spontaneously formed self‐aggregation differ from forced aggregation?
3. Can convection be forced to aggregate through shortwave heating perturbations when key feedbacks required for self‐aggregation are removed, such as spatial gradients in longwave fluxes?

## 2 Methods

### 2.1 Model Set‐Up

In all simulations, the ICOsahedral Nonhydrostatic (ICON) Atmospheric GCM (Giorgetta et al., 2018; Zängl et al., 2015), version 1.8, is used. The ICON GCM model uses the ECHAM6 physics packages (for a full description, see Stevens et al., 2013), including a bulk mass‐flux convection scheme (Nordeng, 1994; Tiedtke, 1989) and cloud cover calculated using the relative humidity (Sundqvist et al., 1989). Other parameterization schemes used are the Lohmann and Roeckner microphysics scheme (Lohmann & Roeckner, 1996) and the gravity wave scheme as described in Stevens et al. (2013).

ICON is run on a triangular grid, based on dividing spherical icosahedron. Grids are defined as RnBk grids, where the original icosahedron edges are divided into *n* equal arcs, which creates $n^{2}$ new triangles. The arcs of these triangles are then bisected *k* times, to recursively subdivide each triangle into 4 smaller triangles. Therefore, the total number of triangular cells in each grid is defined as

(1) $$n_{c}=20n^{2}4^{k}$$

In this study, we employ the R02B04 grid, which has 20,480 cells, with an average cell area of ∼25,000 ${km}^{2}$. This leads to an approximate equivalent grid‐spacing of 160 km. The vertical resolution is set by assigning 47 stretched model levels between the surface and model top at 83 km, with grid spacings ranging from 40 m between the lowest model layers, to around 1,350 m at 15 km, and 5,900 m near the model top.

### 2.2 Experimental Set‐Up

ICON is used in an RCE configuration. The RCE simulations are setup on an aquaplanet with no rotation and no diurnal cycle. The modeling parameters described next are taken from Wing et al. (2018), the RCE model intercomparison project (RCEMIP) to study convective aggregation in radiative‐convective equilibrium models. To initialize the RCE state, homogenized boundary conditions are set where the solar insolation is 551.58 ${Wm}^{-2}$ with a fixed zenith angle of 42.05°. Together these values give a constant insolation of 409.6 ${Wm}^{-2}$. This is equivalent to the annual mean tropical insolation (Wing et al., 2018). The global ocean albedo is set to 0.07. Concentrations of tracers CO_2_, CH_4_, N_2_O and O_2_ are set to be constant in space and time and the O_3_ profile is the same as used in Popke et al. (2013). SSTs are kept globally constant throughout each simulation at either 290K or 305K. The model spin‐up is done for 1 month, with horizontally homogenized radiative fluxes, where the longwave and shortwave cooling rates are averaged horizontally at each model level and timestep. Then the domain‐averaged conditions over the final 5 days of these simulations are used to initialize the simulations, following Wing et al. (2018). Each simulation is then run for 5 years. Model output is given every 5 days, as a 5‐day mean. All timeseries in this paper are therefore plotted for these 5‐day means.

Most simulations have been run with interactive radiation. However to perform the mechanism denial experiments, radiative feedbacks have been turned off by horizontally homogenizing the radiative cooling rates at each model level and timestep, following Muller and Held (2012). Occasionally, we reference a control run at either 290K or 305K SST. In these control simulations, we horizontally homogenize the longwave radiative cooling rates to prevent the aggregation of convection (e.g., Arnold & Putman, 2018; Muller & Held, 2012; Wing & Emanuel, 2014). This enables us to decipher effects of aggregation, by comparing simulations to a non‐aggregated base state.

### 2.3 Aerosol Plume Model

**Figure 1:**
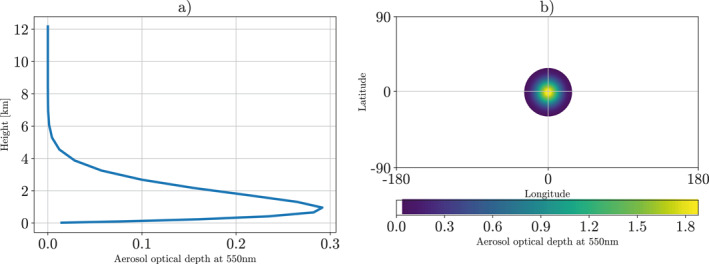
Aerosol plume, centered on latitude, longitude = (0,0), maximum aerosol optical depth (AOD) = 1.8 and single scattering albedo = 0.8, decreasing in a Gaussian way with a standard deviation of 10°. (a) Vertical profile of AOD at the center of the plume. (b) Horizontal extent of the vertically integrated plume AOD, up to three standard deviations. This contains 99.7% of AOD.

The aerosol plume in this study is prescribed using the Max Planck Institute Aerosol Climatology version 2, Simple Plume (MACv2‐SP) model (Stevens et al., 2017). MACv2‐SP is a parameterization used to simplify the representation of anthropogenic aerosol distributions by prescribing the number and features of the aerosol plumes. Parameters to decide include the aerosol optical depth (AOD), single scattering albedo (SSA), and plume spatial extent, and these are given as functions of geographical location, time, and wavelength. In this study, we remove any temporal dependence and use constant parameters for each variable. We also only use a single plume in our simulations, and in order to isolate the role of the heat perturbation we only introduce the radiative properties of the perturbation, not the microphysical effects which are also described in Stevens et al. (2017). In this study, unless otherwise specified, experiments are performed with a single plume at latitude = 0°, longitude = 0°, AOD = 1.8 and SSA = 0.8. This produces a strongly absorbing plume, which causes a large heating perturbation. The horizontal shape of the plumes is modeled with a Gaussian distribution with AOD reducing by a standard deviation every 10° in each direction. Hence, ∼68% of the aerosols lie within a 10° radius of the plume center, and 95% lie within 20°. The vertical distribution of the aerosols is determined by the kernel of Euler's β function with most aerosols occurring below 5 km (for more information, see Stevens et al., 2017). This bottom‐heavy vertical distribution is based on a climatology of aerosols, and ensures the modeled plumes are not unrealistically top‐heavy. The vertical and horizontal distribution of the AOD is shown in Figure 1. It is important to note, realistic absorbing aerosol plumes can reach AODs upwards of 0.6 with SSAs of 0.8 and under (e.g., Sharma et al., 2017), with some places regularly reaching an AOD over 1 (e.g., Filonchyk et al., 2019; Kiely et al., 2019). Therefore, while the plume used here is far stronger than many aerosol plumes seen in the real world, plumes of this strength are seen in certain places (for example, Eastern China, South‐East Africa, and Indonesia).

### 2.4 Metric

Convective aggregation is associated with a moistening of the convective regions, and a drying surrounding them. This therefore causes a broadening of the moisture distribution and, in turn, an increase of the horizontal moisture variance. To compare the degree of aggregation between different simulations in this study we will therefore use the variance of the column‐integrated relative humidity ($\sigma_{CRH}^{2}$) (Wing & Cronin, 2016). In this study, CRH is defined as the density‐weighted integral of relative humidity, integrated from the surface to model top. This removes the temperature dependence of the humidity and enables comparisons of the moisture between different simulations (Wing & Emanuel, 2012). It is important to note that this metric does not have a threshold value to determine when a simulation has aggregated, it is instead used as a comparison tool to determine which atmospheres are more aggregated than others.

### 2.5 Frozen Moist Static Energy Budget

A useful framework for diagnosing aggregation mechanisms is using the FMSE budget equation, defined in Wing and Emanuel (2014). Here, FMSE is defined as

(2) $$h=gz+C_{p}T+L_{v}q_{v}-L_{f}q_{ice}$$

where gz is the geopotential, $C_{p}$ is the specific heat capacity of dry air, T is the temperature, $L_{v}$is the latent heat of vaporization, $q_{v}$ is the water vapor mixing ratio, $L_{f}$ is the latent heat of fusion, and $q_{ice}$ is the ice mixing ratio. We also define the density‐weighted integral of h:

(3) $$\hat{h}=\int h\rho dz$$

integrated over the troposphere, where ρ is the air density. Then, following Bretherton et al. (2005) and Wing and Emanuel (2014), this can be written in budget form to investigate the feedbacks affecting the spatial variance of FMSE:

(4) $$\frac{1}{2}\frac{\partial {\hat{h}}^{\prime 2}}{\partial t}={\hat{h}}^{\prime }SEF^{\prime }+{\hat{h}}^{\prime }NetLW^{\prime }+{\hat{h}}^{\prime }NetSW^{\prime }-{\hat{h}}^{\prime }Adv^{\prime }$$

where SEF stands for surface enthalpy fluxes, which is the sum of the surface latent heat and sensible heat fluxes, NetSW and NetLW are the column shortwave and longwave radiative flux convergences respectively, and Adv is the horizontal divergence of $\hat{h}$. The Adv term is calculated as a residual from the rest of the budget. Primed quantities ${\left[\cdot \right]}^{\prime }$ represent anomalies from the global mean. Each term on the right‐hand side of the budget equation represents a covariance with a source or sink of FMSE. Therefore, a positive term represents a positive feedback of aggregation.

## 3 Unforced Versus Forced Aggregation of Convection

**Figure 2:**
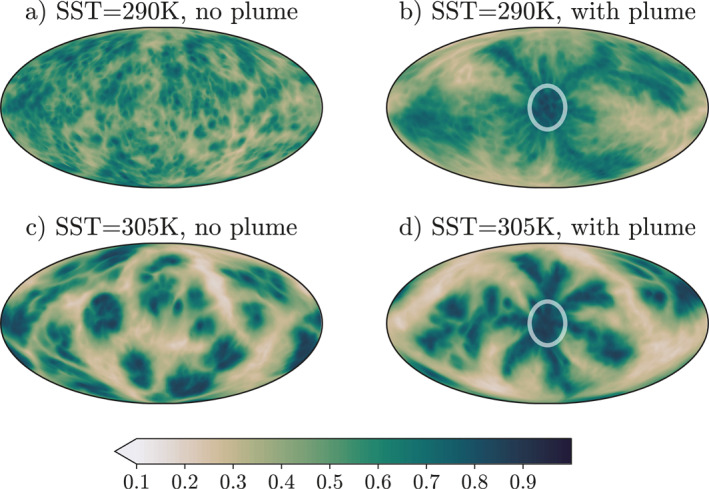
5‐day mean column relative humidity after 5 years of ICON GCM RCE simulations. (a and c) are simulations without an aerosol plume. (b and d) are simulations with an aerosol plume with AOD = 1.8 at the center. White circle shows 20° radius around plume center, containing 95% of AOD. (a–b) sea surface temperature (SST) = 290K, (c–d) SST = 305K.

Here we analyze the model simulations that were run at SSTs of 290K and 305K in RCE with no aerosol forcing and compare them to the model simulations run with the same SSTs, but with the aerosol plume included. Figure 2 shows the 5‐day mean CRH after the five years of the simulations, after equilibrium is reached, for the two different SSTs, 290K and 305K. There are large differences between the amount of aggregation seen in the four simulations. The simulation at 290K without the plume is the least aggregated, as we can see in Figure 2a: the moisture field is the most homogeneous of the simulations, implying the convection is scattered across the globe and there is an absence of large clusters. There is also a lack of large dry patches.

At the higher SST (Figure 2c), there is a more organized moisture field, with extremely wet regions and contrastingly very dry regions. This shows that there are large clusters of deep convection across the globe and this simulation may be classified as aggregated. Through time these clusters are not stationary ‐ the convective clusters do not stay in the same place, and they also grow and decay through time. In contrast to most small domain simulations (e.g., Bretherton et al., 2005; Wing & Emanuel, 2014), the convection does not aggregate into a single large cluster, instead, there are multiple clusters throughout the domain in the aggregated case (Figure 2c). This is, however, consistent with other convective self‐aggregation studies (e.g., Muller & Bony, 2015; Yang, 2019).

When the plume is used in the simulations at 290K and 305K, it causes a large moistening near to it, and the development of some dry patches away from it. This pattern is analogous to the moisture signal convection aggregation causes. The plume forces convection to aggregate around itself. In Figure 2b we see the effect the plume has on the moisture field at an SST of 290K. Comparatively to the simulation at the same SST with no plume, we can see large areas of dry patches and a very moist region near the plume center. Therefore, we expect this simulation to have a larger degree of aggregation to the simulation without a plume. As shown in Figure 2d, using a plume at an SST of 305K converges convection toward it. However, here we do not see a visible difference in the horizontal moisture variance.

**Figure 3:**
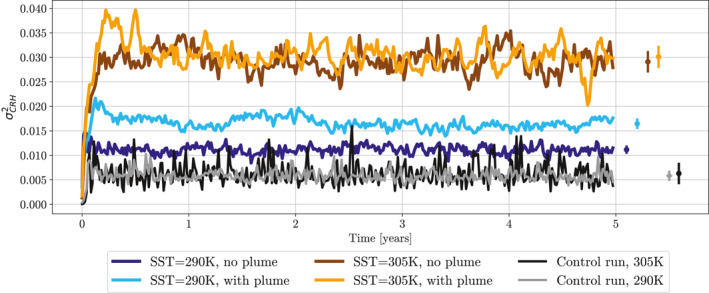
5‐day mean time evolution of variance of column relative humidity for ICON GCM simulations in radiative‐convective equilibrium. Black and gray lines show the control run at 305K and 290K sea surface temperatures (SSTs), respectively. Controls are simulations run with horizontally homogenized longwave heating. Orange and brown lines for simulations at 305K SST with and without a plume, respectively. Light and dark blue lines for simulations at 290K SST with and without a plume, respectively. Circles show mean over the final 4 years of simulation, vertical bars represent one standard deviation of the 5‐day mean $\sigma_{CRH}^{2}$ in time. The aerosol plume has AOD = 1.8 at the center.

These findings are reinforced when looking at timeseries of our aggregation metric, $\sigma_{CRH}^{2}$ in Figure 3. All four simulations have an increased degree of aggregation, when compared to the not‐aggregated control runs. The simulations at 305K with and without the plume are the most strongly aggregated, showing that adding the plume forcing to an already strongly aggregated simulation primarily causes a reorganizing of the aggregated convection and does not force a significant change in the degree of aggregation. At an SST of 290K, we see that, without the plume there is a small increase in the degree of aggregation comparatively to the control run, however the simulation is significantly less aggregated than that at 305K. When using a plume at 290K, we see that the addition of the plume has forced an increase in the degree of aggregation.

**Figure 4:**
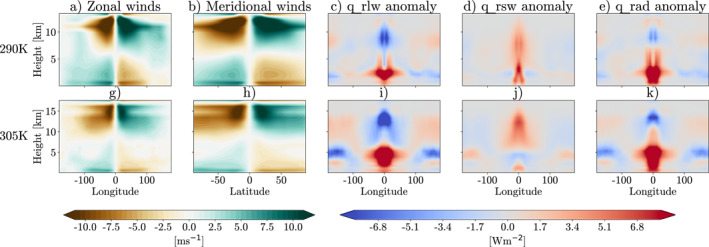
4‐year mean (a and g) Zonal winds, (b and h) Meridional winds, (c and i) longwave heating anomaly, (d and j) shortwave heating anomaly, (e and k) total radiative heating anomaly. Anomalies are taken from the global mean in each simulation. All figures show a slice at latitude = 0°, except (b and h) which show a slice at longitude = 0°. Top row is for simulations at 290K sea surface temperature (SST), bottom row is for simulations at 305K SST. All simulations are from ICON GCM model runs in radiative‐convective equilibrium aerosol plume which has AOD = 1.8 at the center.

The plume model used for these simulations creates large shortwave heating anomalies, which have the ability to generate strong, thermally driven circulations (Dagan et al., 2019). This is the mechanism that is hypothesized to drive the increase in the degree of aggregation seen in these simulations. Figure 4 shows 4‐year mean 3d zonal and meridional wind fields and the longwave, shortwave and total radiative heating horizontal anomalies for the two simulations with the plume. It is clear from Figures 4a, 4b, 4f, and 4g that the plume incites a truly global circulation. There is strong convergence near the surface and strong divergence in the upper atmosphere. This is driven by the radiative anomalies caused by the plume with feedbacks caused by changes in clouds and water vapor. The plume itself incites the large shortwave heating anomalies we can see in Figures 4d and 4i. There are significant differences in the vertical profile of the shortwave heating caused by the plume. At 290K SST this heating is concentrated in the lower troposphere and boundary layer, while at 305K the heating is strongest in the upper troposphere. This could explain why we see an increase in the degree of aggregation at 290K SST, and not at 305K, supported by work by Yang (2018a), who found boundary layer radiative feedbacks were essential for aggregation.

There is also a clear signal in the longwave heating anomalies (Figures 4c and 4h). Here we see a strong negative anomaly in the upper troposphere close to the plume. This is due to the low emission temperature of the deep convective clouds formed around the plume. In the region of the plume, in the boundary layer we see a strong longwave heating. This is due to the absorption and re‐emission of longwave radiation from the bottom of the convective clouds, and the additional absorption from the high amounts of moisture in that area. In the 305K simulation, which is more aggregated than the 290K simulations, we can also see a strongly negative anomaly in the longwave heating at 5 km height, around ±100° longitude. This is displaying the strong longwave cooling in the dry regions of the domain that has been shown to be vital to forming aggregation due to the boundary layer circulation it incites (e.g., Muller & Held, 2012; Sessions et al., 2016, and others).

**Figure 5:**
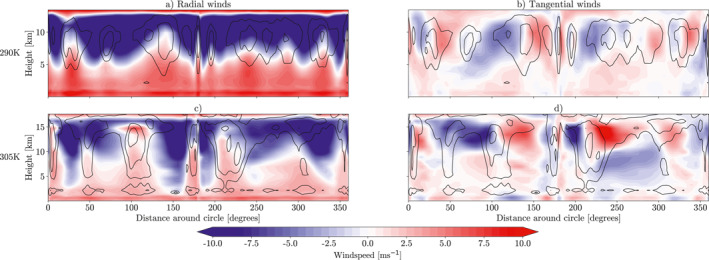
5‐day mean slices of wind field taken at a radius of 40° around the plume center. The radial (a and c) and tangential (b and d) fields have then been calculated. Cloud fraction contours are shown in black at (0.15, 0.4, 0.65). Positive radial winds are moving inwards, toward the plume. Positive tangential winds are moving clockwise around the plume. Top row is for simulations at 290K sea surface temperature (SST), bottom row is for simulations at 305K SST. All simulations are from ICON GCM model runs in radiative‐convective equilibrium with an aerosol plume with AOD = 1.8 at the center.

Figure 5 shows slices of winds taken around a 40° radius circle, centered on the plume's maximum AOD (latitude, longitude = 0°). Radial and tangential wind contributions have then been calculated for each of the simulations with a plume at 290K and 305K SST. In the radial wind fields (Figures 5a and 5c) the large‐scale circulation can be seen. These radial winds show that there is generally convergence toward the plume center near the surface and divergence in the upper troposphere away from the deep convection. This is consistent with the thermally driven circulation that was shown by Dagan et al. (2019) and that can be seen in Figure 4. In the places where there is deep convection, the convergence extends higher into the atmospheric column, particularly prevalent in the simulation at 305K SST (Figure 5c). This implies the convection is converging toward the plume at all heights. Hence, as convection pops up randomly some distance from the plume this will then converge toward the plume, and cluster together with the existing convection. This can sometimes cause aggregation to have radial branches extending out of the central cluster, for example, in Figure 2d. This aggregated spatial distribution can also be seen in other aggregation simulations in GCMs, such as Coppin and Bony (2015), Figure 1. The winds working tangentially to the plume also show a secondary circulation. This circulation has tangential surface convergence toward the radial convection branches and upper atmospheric divergence. This circulation helps to organize convection into lines connected to the central cluster around the plume, and helps to maintain areas of drier, clear sky in‐between these lines.

### 3.1 Assessment of Hysteresis

Many previous studies of convective self‐aggregation have found the convective cluster to exhibit hysteresis ‐ that is, once it has aggregated it will remain so even if the necessary feedbacks are removed (e.g., Khairoutdinov & Emanuel, 2010; Muller & Held, 2012). However, in the simulation at SST = 290K with the aerosol forcing, the aggregation has been forced to occur through temperature perturbations creating thermally driven circulations. This aggregation is not necessarily maintained through the usual feedback interactions. To investigate whether the circulations driving this organization are self‐sustaining and therefore able to maintain the aggregation when the plume is not there anymore, we ran a simulation where the plume is removed after 5 years at SST = 290K. We then compare the $\sigma_{CRH}^{2}$ at the end of this simulation, the simulation with no plume and the original 5‐year simulation with the plume. We found that, after the plume has been removed, $\sigma_{CRH}^{2}$ drops back down to a value comparable to the simulation without a plume (not shown). Therefore, the aerosol plume does not generate convective aggregation that exhibits hysteresis, and the existence of the cluster is directly linked to the existence of the forcing.

### 3.2 Sensitivity to Aerosol Plume Strength

A number of sensitivity experiments were performed to investigate the results' robustness for different aerosol radiative perturbations. A range of 7 different AODs were used, including the original AOD = 1.8 and these were all compared with the experiment with no plume. The other AODs tested were 0.3, 0.4, 0.5, 0.6, 1, and 1.2. To generalize these results to different combinations of AOD and SSA, we discuss the plume ‘strength’ with respect to the aerosol absorption optical depth (AAOD), which is defined as

(5) AAOD=AOD*(1−SSA)

Therefore, the values of AAOD tested were 0.06, 0.08, 0.1, 0.12, 0.2, 0.24, and 0.36.

**Figure 6:**
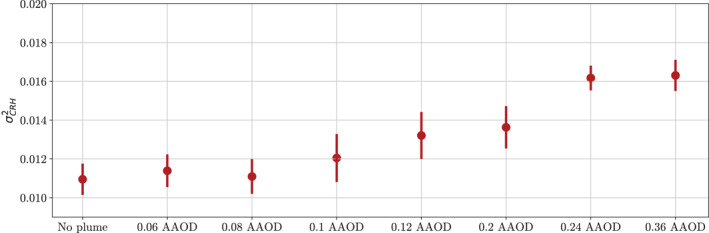
4‐year mean variance of column relative humidity for simulations run at 290K. Vertical lines show one standard deviation around the means. All simulations are from ICON GCM model runs in radiative‐convective equilibrium with an aerosol plume (except for No plume), centered on latitude, longitude = (0,0), and single scattering albedo = 0.8. Simulation plume maximum absorption aerosol optical depth at center increases from 0.06 to 0.36 along the *x*‐axis.

As AAOD is increased, the degree of aggregation also generally increases, until an AAOD of 0.24 (Figure 6). The three simulations with the weakest forcings (AAOD = 0, 0.06, 0.08) do not show much difference in their $\sigma_{CRH}^{2}$. When the AAOD is increased to 0.12 or greater there are significant increases in $\sigma_{CRH}^{2}$ and therefore, increases in the degree of aggregation. The simulations with an aerosol plume AAOD of 0.24 and 0.36 show the strongest response, with the simulations with an AAOD of 0.12, and 0.2 also showing an increase in $\sigma_{CRH}^{2}$ compared with the simulation without an aerosol plume. From this, we note that, for the aerosol plume to have an impact on the aggregation convection at an SST of 290K, there must be an AAOD of at least 0.12. Pollution plumes seen in the real‐world can have AAODs of 0.12 or greater during high pollution events, such as the during biomass burning events (e.g., Sharma et al., 2017), or dust events (e.g., Filonchyk et al., 2020), and so we hypothesize that these aerosol plumes could have a local impact on convective organization.

## 4 Physical Mechanisms Behind Forced Aggregation

### 4.1 Frozen Moist Static Energy Budget

Convective aggregation has been shown to be dependent on radiative feedbacks many times (e.g., Arnold & Putman, 2018; Coppin & Bony, 2015; Muller & Held, 2012; Yang, 2018a). Interactions between longwave fluxes, humidity, and convection are generally found to be essential for the triggering of aggregation. Shamekh et al. (2020) found that when they forced convection to aggregate, using an SST hotpot, this induced enough convective instability on its own, that aggregation occurred in the absence of the usually necessary longwave feedbacks. In this section, we investigate, using the frozen moist static energy (FMSE) budget described in Section 2.5 (Wing & Emanuel, 2014), the physical mechanisms driving the aggregation of convection in our simulations. In particular, we are interested in how the relative contributions from the shortwave feedbacks differ when using the plume to force aggregation.

**Figure 7:**
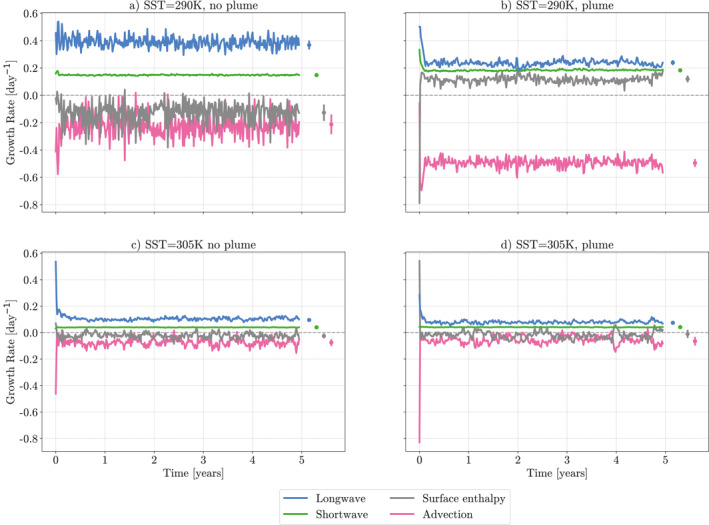
5‐day mean time evolution of domain mean feedback growth rates in the frozen moist static energy budget. (a and c) simulations at sea surface temperature (SST) of 290K, (b and d) SST at 305K. Circles show final 4‐years mean, with vertical line showing one standard deviation of the 5‐day mean feedback growth rates in time. All simulations are from ICON GCM model runs in radiative‐convective equilibrium. (b and d) are run with an aerosol plume with AOD = 1.8 at the center. Gray dotted line highlights *y* = 0.

**Figure 8:**
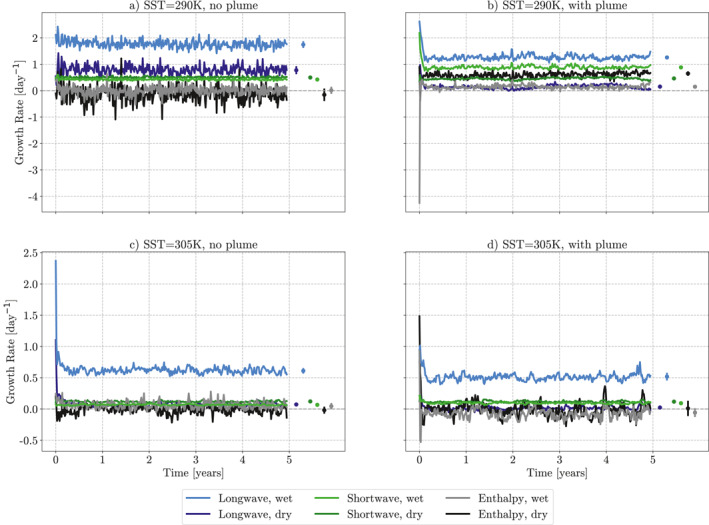
5‐day mean time evolution of mean feedback growth rates in the frozen moist static energy budget in the wettest 5% of columns (lighter lines) and driest 5% of columns (darker lines). (a and c) simulations at sea surface temperature (SST) of 290K, (b and d) SST at 305K. Circles show final 4‐years mean, with vertical line showing one standard deviation of the 5‐day mean feedback growth rates in time. All simulations are from ICON GCM model runs in radiative‐convective equilibrium. (b and d) are run with an aerosol plume with AOD = 1.8 at the center. Gray dotted line highlights *y* = 0.

Figure 7 compares the relative contributions of the $\hat{h}NetLW^{\prime }$, $\hat{h}NetSW^{\prime }$, $\hat{h}SEF^{\prime }$, and $\hat{h}Adv^{\prime }$, feedbacks to the overall growth rate of the FMSE variance, from Equation 4 for the simulations at 290K and 305K SSTs, both with and without the plume. Hereafter, these feedbacks are referred to as the longwave, shortwave, surface enthalpy, and advective feedbacks, respectively. It is clear that in each simulation the strongest, positive relative contribution comes from the longwave feedbacks once the simulations have reached equilibrium. This follows previous studies, such as Coppin and Bony (2015), Arnold and Putman (2018). In the three, more aggregated, simulations (290K with the plume, 305K with and without the plume) the contribution from the longwave feedbacks decreases after the initial aggregating period, but still remains the strongest relative feedback. When the contribution is split into the contribution from the wettest 5% and driest 5% of columns (Figure 8) we can see this strong longwave feedback is primarily working in the wettest regions of the domain. These wet regions correlate with the cloudiest regions in the domain. In the aggregating stage of the simulations, the relative contribution from the longwave feedback also dominates for all simulations except in 305K with the plume (Figure 7d).

The relative contribution from the shortwave feedbacks is strongest in the simulation at an SST of 290K, with the plume (Figure 7b). Here, there is an almost equal contribution from the longwave and shortwave feedbacks. This increase comes from the inclusion of the plume, driving a large positive perturbation in the shortwave convergence. Interestingly we do not see the same dominance from the shortwave feedbacks in the simulation with the plume at 305K SST (Figure 7d). A theory for this is given in the Section 4.1.2. The driver of this increase in the shortwave contribution comes mainly from the wettest columns of the domain, with the contributions in the driest columns remaining mostly equal to those in the simulation at 290K with no plume.

At an SST of 305K, the contribution from the surface enthalpy fluxes is close to zero, however, at 290K we see a negative contribution in the simulation without a plume, and a positive contribution in the simulation with a plume. The sign of the enthalpy feedback is driven by the feedbacks in the driest columns in both these simulations. Moreover, it is also driven mostly by the latent heat fluxes in these dry columns (not shown). It is hypothesized that the strongly positive feedback in the simulation with the plume is due to increased windspeed due to the increased convergence driven by the plume. This is the positive WISHE mechanism described in Coppin and Bony (2015). For the remainder of this paper, we will focus on the radiative feedbacks due to the direct interactions between the plume and radiative feedbacks.

#### 4.1.1 Longwave Feedbacks

The key positive feedback behind the longwave mechanism in these simulations is the strong longwave warming in the boundary layer below the clouds, combined with the low emission temperatures at the top of the deep convective clouds. This causes a strong temperature gradient, driving a humidity gradient and an upgradient transport of FMSE. The cloud driven feedback is the same as the longwave maintenance feedback described in Wing and Emanuel (2014).

#### 4.1.2 Shortwave Feedbacks

The small, but positive, shortwave feedbacks in the plume‐free runs are simple to understand: as water vapor is an absorber in the shortwave, wet columns will have higher absorption than the drier columns. This correlates well with the anomalies of $\hat{h}$, and so we see a positive shortwave feedback everywhere in the domain. The increased contributions from the shortwave feedbacks in the wettest columns in the run at 290K SST with the plume arise from the direct shortwave absorption associated with the plume. This strong shortwave perturbation, also seen in Figure 4d, is the driving influence of the increased degree in aggregation for this simulation. As stated before, we do not see the same increase in the shortwave contribution with the plume at 305K SST. This is likely due to the fact that, at this SST, the longwave feedbacks are sufficient to drive aggregation, as proven by the highly aggregated simulation at 305K SST without the plume. Therefore, the introduction of the shortwave perturbation at this SST drives a thermally driven circulation, which influences the shape in which convection aggregates, but is not required for the aggregation itself.

### 4.2 Mechanism Denial Test

To further investigate the role longwave and shortwave feedbacks play with and without the plume in our simulations, we ran a suite of experiments where the longwave and shortwave fluxes were homogenized in turn at each timestep, and at each model layer, following Muller and Held (2012). Shamekh et al. (2020) found that when they forced convection to aggregate, using an SST hotpot, this induced enough convective instability on its own, that aggregation occurred in the absence of the usually necessary longwave feedbacks. Here, we are particularly interested in whether our convection can also be forced to aggregate in the absence of the longwave feedbacks.

In this section, we describe simulations by SST_plume_homogenization, so the experiment at 290K SST, without a plume and with longwave fluxes homogenized would be called 290K_homogLW, and the run with the same SST and homogenization but with the plume would be called 290K_plume_homogLW. We also note that the 290K_plume_homogLW experiment has a plume with AOD = 1, and the 305K_plume_homogLW experiment has a plume with AOD = 0.8, compared to the plume with AOD = 1.8 used in the rest of the simulations. However, testing has shown that a plume of this shape and size, with SSA = 0.8, can force an increase in the degree of aggregation at AODs of 0.6 or greater. Also, these two simulations homogenize the longwave fluxes for three out of every four timesteps. Whilst this does not fully homogenize the longwave heating field, we have seen that this amount of homogenizing is sufficient to decrease the degree of aggregation to close to the value in our control runs (not shown). These measures are taken to ensure that the model stays in equilibrium as using high AOD and fully homogenizing the longwave heating leads to excessive instabilities.

#### 4.2.1 Unforced Simulations

**Figure 9:**
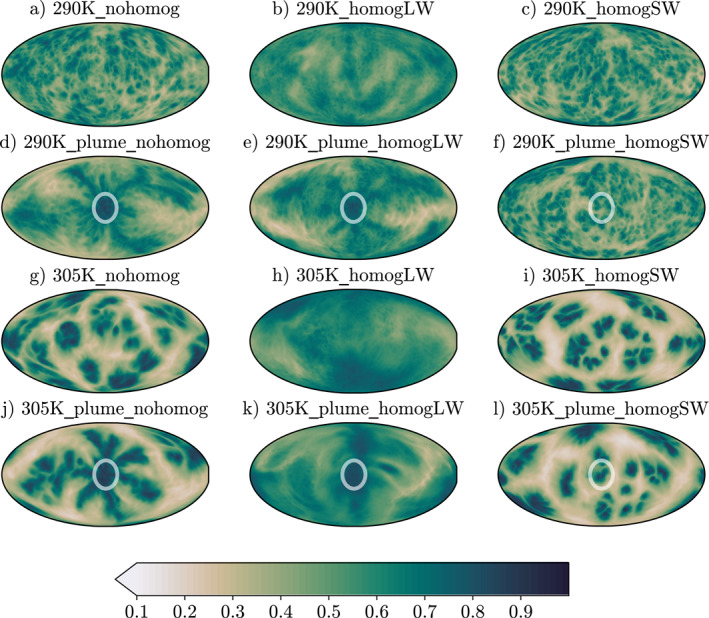
5‐day mean column relative humidity after 5 years of ICON GCM RCE simulations. (a–c) and (g–i) are simulations without an aerosol plume. (b–d) and (j–l) are simulations with an aerosol plume, centered inside the white circle, maximum aerosol optical depth (AOD) = 1.8 (except e) which has AOD = 1, and (k) which has AOD = 0.8) and single scattering albedo = 0.8, decreasing in a Gaussian way with a standard deviation of 10°. White circle shows 20° radius around plume center, containing 95% of AOD. (a–f) Sea surface temperature (SST) = 290K, (g–l) SST = 305K. First column shows experiments with fully interactive radiative heating, second column shows experiments with longwave heating rates horizontally homogenized at each model level, third column shows experiments with shortwave heating rates horizontally homogenized at each model level. All homogenizing is done at every timestep, except for (e) 290K_plume_homogLW and (k) 305K_plume_homogLW, where homogenizing is done for 3 out of every 4 timesteps.

**Figure 10:**
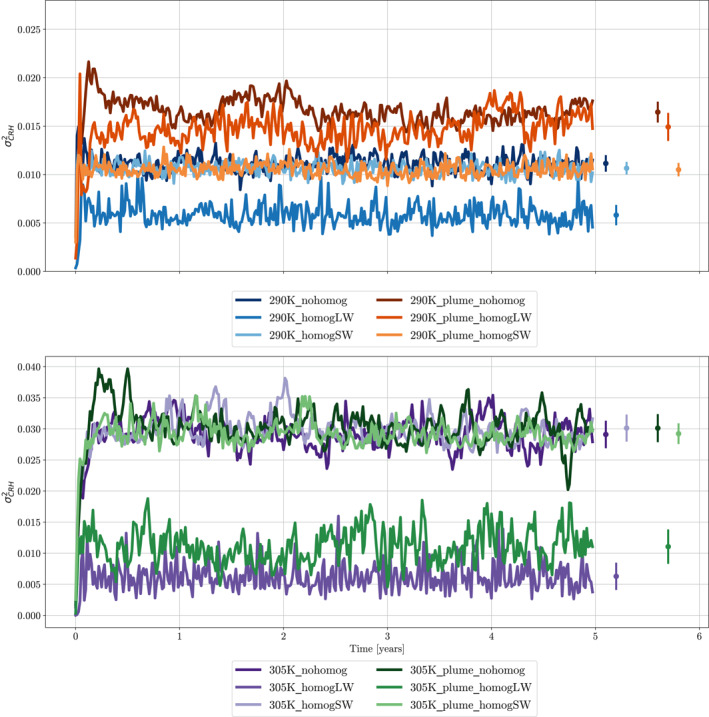
5‐day mean time evolution of variance of column relative humidity for ICON GCM simulations in radiative‐convective equilibrium. Top/bottom panel shows simulations at SST = 290K/305K. Blue and purple lines show simulations with no plume, orange and green lines show simulations with aerosol plume. Darkest line in each color shows simulation with fully interactive radiation, second darkest line shows simulations with homogenized longwave fluxes, lightest lines show simulations with homogenized shortwave fluxes. Circles show mean over the final 4 years of simulation, vertical bars represent one standard deviation of the 5‐day mean $\sigma_{CRH}^{2}$ in time. Aerosol plume has AOD = 1.8 at the center. All homogenizing is done at every timestep, except for (e) 290K_plume_homogLW and (k) 305K_plume_homogLW, where homogenizing is done for 3 out of every 4 timesteps.

Figure 9 compares the CRH at the end of each 5‐year simulation across this suite of experiments. First, focusing on the simulations without a plume, we can see that homogenizing the longwave fluxes removes all aggregation, leaving a mostly homogeneous moisture field (Figures 9b and 9h). This is expected, given the longwave feedbacks dominate in all simulations, discussed in the previous section. This is also in line with previous studies, such as Muller and Held (2012), Yang (2018a), Beucler and Cronin (2019) and others. To further prove that the removal of interactive longwave fluxes removes aggregation we can compare the timeseries of our metric, $\sigma_{CRH}^{2}$, for runs with fully interactive fluxes and homogenized longwave fluxes. It is clear, from Figure 10, that the removal of interactive longwave fluxes significantly reduces the degree of aggregation at both 290K and 305K SST.

When shortwave fluxes are homogenized, there is little visible difference in how aggregated the moisture field is. Indeed, when the degree of aggregation is compared in Figure 10, we can see that, at both 290K and 305K SST there are no significant changes in $\sigma_{CRH}^{2}$.

#### 4.2.2 Forced Simulations

When shortwave fluxes are homogenized with the plume in the simulations, the moisture distribution returns to roughly how it was arranged with no plume. This makes intuitive sense, as our plume model works by perturbing the shortwave absorption. When these perturbations are then homogenized across the domain, we will no longer see spatial evidence of the plume. We can see this in Figures 9f and 9l. Similarly, the degree of aggregation in these runs is equal to the equivalent runs without a plume, (Figure 10).

The key remaining question, is whether the shortwave perturbations induced by the plume generate a powerful enough circulation to force convection to aggregate without interactive longwave fluxes. From the evidence provided in Section 4.1, one might expect that we see a different response to homogenizing longwave fluxes at 290K and 305K, due to the smaller shortwave contribution at 305K. First, by visual inspection of the horizontal moisture maps in Figures 9e and 9k this appears to be the case. In 305K_plume_homogLW (Figure 9k) we can see only a small amount of evidence of the plume. Here, the homogenization of the longwave fluxes has prevented most of the aggregation. This is further supported in Figure 10, where the degree of aggregation in 305K_plume_homogLW is far lower than that in 305K_nohomog, and only slightly higher than the degree of aggregation in 305K_homogLW, our original control case. In the 290K_plume_homogLW experiment, there is significantly more visual evidence of the plume aggregating convection in Figure 9e. Inspection of our metric timeseries in Figure 10 shows that, 290K_plume_homogLW has a higher degree of aggregation comparatively to 290K_nohomog. This supports results from Shamekh et al. (2020), who also found that they could force convection to aggregate using SST hotspots even with homogenized radiative heating. However, we still see a slight decrease in the degree of aggregation when the longwave fluxes are homogenized compared to 305K_plume_nohomog. This implies that, whilst not always a necessary feedback, the longwave feedbacks systematically aid the aggregation of convection with the plume and that the shortwave heating induced by the plume is not the only driver of the thermally driven circulation. The strong boundary‐layer warming in the longwave caused by the aggregation of moisture and convection around the plume is also important for maintaining the circulation.

**Figure 11:**
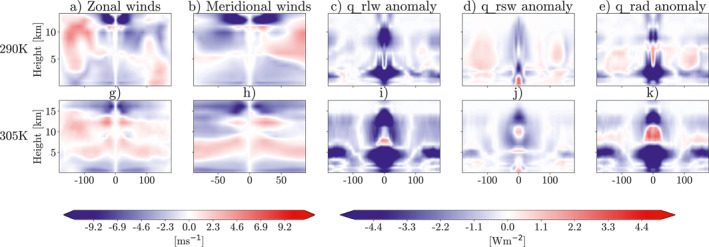
4‐year mean difference between the absolute magnitude of (a and g) Zonal winds, (b and h) Meridional winds, (c and i) longwave heating anomaly, (d and j) shortwave heating anomaly, (e and k) total radiative heating anomaly for simulations with horizontally homogenized longwave radiation minus fully interactive radiation (e.g., homogLW ‐ nohomog). All figures show a slice at latitude = 0, except (b and h) which show a slice at longitude = 0. Top row is for simulations at 290K sea surface temperature (SST), bottom row is for simulations at 305K SST. All simulations are from ICON GCM model runs in radiative‐convective equilibrium with an aerosol plume, centered on latitude, longitude = (0,0), and single scattering albedo = 0.8. Nohomog simulations both have maximum aerosol optical depth (AOD) of 1.8 at the plume center, homogLW at 290K has maximum AOD = 1, homogLW at 305K has maximum AOD = 0.8.

To fully understand why the shortwave feedbacks are more effective at aggregating the convection at 290K SST with the plume (compared to 305K SST) we investigate the differences in the absolute magnitude of the circulation and radiative heating perturbations induced by the plume when longwave fluxes are homogenized (Figure 11). At both SSTs there is an increase in the shortwave heating perturbation near the surface when longwave fluxes are homogenized, most likely due to a reduced cloud shielding effect. The magnitude of this shortwave anomaly is greater at 290K SST than at 305K. This leads to a weaker reduction in the thermally driven circulation at 290K. This means that, even with homogenized longwave feedbacks causing a large reduction in boundary layer longwave heating near to the plume, the thermally driven circulation is being controlled mainly by the shortwave feedbacks at 290K SST. The boundary layer shortwave heating in 290K_plume_homogLW, is therefore managing to drive some aggregation and so is responsible for the increase in the degree of aggregation between 290K_nohomog and 290K_plume_homogLW in Figure 10. At the higher SST, 305K, there is some surface shortwave heating near to the plume in Figure 11j, however, this is extremely weak. We hypothesize that this is the reason for the increase in the degree of aggregation between the 305K control run and 305K_plume_homogLW being smaller than the increase between the 290K control run and 290K_plume_homogLW (Figure 10).

## 5 Conclusions and Implications

Convective self‐aggregation has been shown to occur in many different modeling setups, including in general circulation models (GCMs) with a convective parameterization scheme. Work thus far has focused on spontaneously occurring self‐aggregation. In this study, we focus on convective aggregation in the presence of an external source of diabatic heating, representative of aerosol radiative perturbations. This plume induces a thermally driven circulation. We were particularly interested in how the forcing affected the ability of a simulation to aggregate, and whether the mechanisms behind this forced aggregation differ from those causing a spontaneous self‐aggregation.

The radiative perturbation caused by the aerosol plume forces an increase in the degree of aggregation through the generation of a large‐scale circulation. This circulation is driven by strong shortwave warming due to the aerosol absorption, and enhanced by strong boundary layer longwave warming from the aggregation of moisture. The aggregation that is forced by this radiative plume is a superposition of two circulations. One circulation has radial convergence toward the plume in the lower atmosphere and radial divergence in the upper atmosphere. The second circulation works tangentially to the plume again with lower‐level convergence and upper‐level divergence, shaping the lines of convection connected radially to the central cluster formed around the plume.

The forced aggregation shown in this study is dependent on the aerosol plume. When the plume was removed, the convection immediately disaggregates. Therefore, in contrast to previous studies (e.g., Khairoutdinov & Emanuel, 2010; Muller & Held, 2012), forced aggregation does not exhibit hysteresis and is entirely dependent on the plume forcing's existence in this study.

Longwave feedbacks are found to be essential for forming convective self‐aggregation, in line with many previous studies (e.g., Arnold & Putman, 2018; Muller & Held, 2012; Wing & Emanuel, 2014) at both SSTs, however, at lower SSTs, in the presence of a strong shortwave radiative heating perturbation convection can be forced to aggregate in the absence of longwave feedbacks. In addition, the contribution from surface enthalpy flux feedbacks varies at different SSTs, with a stronger contribution at lower SSTs. When comparing the mechanisms behind unforced and forced aggregation, we find that shortwave feedbacks play a larger role in forming the aggregation at 290K SST with the plume than any other simulation. This is confirmed through mechanism denial experiments with horizontally homogenized longwave radiative heating, where convection is able to aggregate through the induced circulation, driven by shortwave heating anomalies.

We have shown that an aerosol absorption optical depth (AAOD) of 0.12 or greater is required to force simulations to aggregate at an SST of 290K, and the maximum degree of aggregation is reached at an AAOD of roughly 0.24. There are many examples of aerosol plumes with AAODs greater than 0.12 in the real world such as pollution, dust events, and biomass burning events (e.g., Eck et al., 2003; Filonchyk et al., 2020; Sharma et al., 2017; Vadrevu et al., 2015). Therefore, these events could be an important real‐world analogue for convective aggregation. However, the real atmosphere contains far more complexity than is included in the model used here. Features of this complexity, such as planetary rotation, land, and an interactive sea surface, can hugely affect the lifetime and size‐spectrum of convection. Therefore, we can hypothesize that these dust and pollution events may have an effect on locally organizing convection, but more work is needed to deduce how this may be manifested and what feedbacks are working in the real atmosphere.
